# Supplementary material for: PIM1 promotes hepatic conversion by suppressing reprogramming-induced ferroptosis and cell cycle arrest
Source: Nat Commun. 2022 Sep 6;13:5237. doi: 10.1038/s41467-022-32976-9 (PMC9448736; doi:10.1038/s41467-022-32976-9)
Supplement: Supplementary file 1 — Supplementary Information [file 41467_2022_32976_MOESM1_ESM.pdf]

Supplementary Information

PIM1 promotes hepatic conversion by overcoming reprogramming-induced ferroptosis and cell cycle arrest

Yangyang Yuan[1,3,7,8], Chenwei Wang[2,8], Xuran Zhuang[3,8], Shaofeng Lin[2,8], Miaomiao Luo[1,8], Wankun Deng[2], Jiaqi Zhou[2], Lihui Liu[1], Lina Mao[1], Wenbo Peng[3], Jian Chen[4,5], Qiangsong Wang[1], Yilai Shu[4,5,*], Yu Xue[2,6,*], and Pengyu Huang[1,3,*]

[1]Institute of Biomedical Engineering, Chinese Academy of Medical Sciences and Peking Union Medical College, Tianjin, 300192, China

[2]MOE Key Laboratory of Molecular Biophysics, Hubei Bioinformatics and Molecular Imaging Key Laboratory, Center for Artificial Intelligence Biology, Institute of Artificial Intelligence, College of Life Science and Technology, Huazhong University of Science and Technology, Wuhan 430074, Hubei, China

[3]School of Life Science and Technology, ShanghaiTech University, Shanghai, 201210, China

[4]ENT institute and Department of Otorhinolaryngology, Eye & ENT Hospital, State Key Laboratory of Medical Neurobiology and Institutes of Biomedical Sciences, Fudan University, Shanghai, 200031, China

[5]NHC Key Laboratory of Hearing Medicine, Fudan University, Shanghai, 200031, China

[6]Nanjing University Institute of Artificial Intelligence Biomedicine, Nanjing, Jiangsu 210031, China

[7]Current affiliation: Centre for Translational Stem Cell Biology Limited., Hong Kong 999077, China

[8]These authors contributed equally: Yangyang Yuan, Chenwei Wang, Xuran Zhuang, Shaofeng Lin, Miaomiao Luo

*Correspondence: huangpengyu@yeah.net (P.H.), xueyu@hust.edu.cn (Y.X.), yilai_shu@fudan.edu.cn (Y.S.)

Contents

Supplementary Note 1. Evaluation of the accuracy of CKI 3
Supplementary Note 2. Additional experiments and analyses of hepatic reprogramming 5
Supplementary Note 3. Additional analyses of the CKI accuracy 7
Supplementary Figures 10
Supplementary References 31

Supplementary Note 1. Evaluation of the accuracy of CKI

In 2016, Zanotto-Filho et al. profiled the transcriptomic changes of a human breast cancer cell line (MDA-MB231) and osteosarcoma cell line (U-2 OS) using a number of chemotherapeutic agents including DOX (1 μM/L, 8 h), and uncovered a regulatory role of nuclear factor erythroid 2-related factor 2 (NFE2L2/NRF2) in chemotherapy resistance[1]. In another study, Hogrebe *et al*. quantified the phosphoproteomes of U-2 OS cells with or without DOX treatment (5 μM/L, 2 h)[2]. From these two studies[1,2], we identified 2197 DEMs and obtained quantitative data for 27,590 p-sites in 5592 proteins (Supplementary Fig. 1a, b). In the phosphoproteomic data, there were 22,744 phosphoserine residues (pS, 82.44%), 4451 phosphothreonine residues (pT, 16.13%), and 395 phosphotyrosine residues (pY, 1.43%) (Supplementary Fig. 1c).

From the literature, known PKs involved in DOX resistance were curated (Supplementary Data 1). Using this independent testing dataset, we evaluated the accuracy of CKI by calculating the AUC score of the method that incorporated all three types of data (mRNA expression, substrate p-site intensity, and kinase-substrate network) and of models built with each data type alone (Supplementary Fig. 1d). We also compared to a *z*-score-based method, Kinase-Substrate Enrichment Analysis (KSEA), which predicts potentially important PKs only from phosphoproteomic data[3,4]. We found that using only data related to changes in substrate p-site intensity or the kinase-substrate network had a comparable accuracy to that of KSEA (AUC = 0.7635, 0.8058, and 0.7871, respectively) (Supplementary Fig. 1d). However, when the three types of data were integrated with CKI, the AUC value was higher than the other methods (0.8278) (Supplementary Fig. 1d). The confusion matrix and principal component analysis (PCA) indicated that known and unknown PKs could be separated in a reasonably accurate manner (Supplementary Fig. 1e, f). Of the

final 22 PKs predicted by CKI, 10 had already been reported as truly important for DOX resistance in U-2 OS cells, including ATM/ATR[5], MAPK13[6], and CHEK1[7] (Supplementary Fig. 1g and Supplementary Data 1). In this case, the transcriptomic data were less informative and only contributed to prioritization of one of the 22 final candidate PKs (Supplementary Fig. 1g and Supplementary Fig. 2a). In addition, we mapped the quantified p-sites to the 22 PKs where available, and found that only one PK activity-associated p-site, ATM S1981[8], was up-regulated after DOX treatment (Supplementary Fig. 1g, Supplementary Fig. 2b and Supplementary Data 1).

Next, we integrated the transcriptomic and phosphoproteomic data of breast cancer cells treated with or without genistein from previous reports[9,10]. In total, we identified 3264 DEMs and obtained quantitative data for 17,403 p-sites in 7149 proteins (Supplementary Fig. 1h-j). Using known genistein-associated PKs as the testing data, the AUC value of CKI was calculated as 0.7912, showing a much higher accuracy than all of the other methods tested (Supplementary Fig. 1k). Again, the results from the confusion matrix and PCA supported CKI could distinguish between known and unknown PKs (Supplementary Fig. 1l, m). In this case, the transcriptomic data also contributed to prioritizing five of the 21 final candidate PKs (Supplementary Fig. 1n, Supplementary Fig. 2c and Supplementary Data 1). No PK activity-associated p-sites were identified to be differentially regulated after genistein treatment (Supplementary Fig. 2d and Supplementary Data 1).

Because both DOX and genistein are potent DNA damage inducers[1,2,9,10], it was not surprising that the major PK known to respond to DNA damage, ATM, was successfully predicted as a central PK in both cases (Supplementary Fig. 1g, n). Taken together, the results showed a promising performance of CKI in well-studied drug-resistant cancer cells.

Supplementary Note 2. Additional experiments and analyses of hepatic reprogramming

We induced hepatic reprogramming by introduction of *FOXA3*, *HNF1A*, and *HNF4A* (FHH) to human dermal fibroblasts (HDFs) as previously described[11]. The expression of FHH were detected within 1 day after lentiviral infection (Supplementary Fig. 4a). Liver-specific functional genes were induced shortly after 2 days' infection. Then the expression of liver-specific genes gradually increased afterwards (Supplementary Fig. 4b). Treatments with staurosporine, a broad-spectrum protein kinase inhibitor, or genistein, a protein-tyrosine kinase inhibitor and potent DNA damage inducer, significantly suppressed hepatic reprogramming of HDFs, suggesting the critical roles of protein kinases (PKs) in hepatic reprogramming (Supplementary Fig. 4c, d).

From the transcriptomic profiling, we quantified 27,593, 25,755, and 27,040 mRNAs in GFP, FHH-2.25d, and FHH-5d HDFs, respectively (Supplementary Fig. 5a and Supplementary Data 3). A similar distribution of fragments per kilobase of exon per million fragments mapped (FPKM) values across different samples indicated that enforced expression of hepatic transcription factors (TFs) did not significantly affect the overall transcriptomic expression during the early stage of hepatic conversion (Supplementary Fig. 5j). To identify differentially regulated phosphorylation sites (p-sites) (DRPs), a factor of 2 ($\geq$ 2-fold or $\leq$ 0.5-fold change) was employed to pairwise compare phosphoproteomes. In total, we obtained 92, 527, and 280 DRPs from the comparisons of FHH-2.25d *vs*. GFP, FHH-5d *vs*. GFP, and FHH-5d *vs*. FHH-2.25d, and found that most of them were markedly changed after 2.25 days' infection (Supplementary Fig. 5k and Supplementary Data 3). Again, the similar distribution of p-site intensities in the three samples indicated that FHH infection did not influence the whole phosphorylation level (Supplementary Fig. 5l).

Next, we compared differentially expressed mRNAs (DEMs) to phosphoproteins containing at least one DRP, and observed a poor overlap

between regulated transcriptomes and phosphoproteomes (Supplementary Fig. 5m). Then we performed the functional enrichment analyses of DEMs and DRPs, using the annotations of Gene Ontology (GO) biological processes. A number of hepatic-associated biological processes such as cellular glucuronidation (GO:0052695), xenobiotic glucuronidation (GO:0052697), and flavonoid glucuronidation (GO:0052696), as well as the cell division (GO:0051301) process, were statistically up-regulated at the transcriptional level (Supplementary Fig. 5o). Interestingly, we observed several viral response processes were transcriptionally activated, possibly due to the lentivirus-based delivery of FHH to HDFs (Supplementary Fig. 5o). In the phosphorylation level, much more diverse signaling processes were enriched (Supplementary Fig. 5p). The poor overlap between enrichment processes of transcriptomic and phosphoproteomic data suggested that distinct biological processes are preferentially up-regulated by transcription or phosphorylation during the early stage of hepatic conversion. Thus, integration of transcriptomic and phosphoproteomic data could reveal much more information of the dynamics of cellular signaling pathways.

Moreover, we mapped the quantified p-sites to the 15 PKs if available, and only found two p-sites of S1773 and S1374 in KALRN and ROCK2, respectively, and neither exhibited a ≥ 2-fold change upon FHH infection (Supplementary Fig. 5r). Of note, none of the two p-sites were associated with their corresponding PK activities. Thus, it was not possible for us to characterize differentially activated or inhibited PKs directly from quantified p-sites. To further evaluate the reliability of the prediction, FPKM values and p-site intensities were randomly shuffled for all transcriptomic and phosphoproteomic data sets, respectively, and potential central PKs were re-calculated by CKI. Such a permutation test was repeated 10,000 times, and all predicted central PKs achieved a false positive rate (FPR) value < 0.01 (Supplementary Data 4).

In addition, we analyzed the phosphoproteomic data using KSEA[3,4] to predict 11 potentially functional PKs involved in hepatic reprogramming, while

the two newly identified PKs were not included (Supplementary Fig. 5s).

Supplementary Note 3. Additional analyses of the CKI accuracy

To evaluate the accuracy of CKI using p-sites with or without normalization by their corresponding protein expression levels, we additionally conducted quantitative proteomic profiling for mouse hepatocyte maturation and human hepatic reprogramming, respectively. For the former, three biological replicates were prepared for immature hepatocytes generated from liver progenitor cells (CLiP-Hep) and mature hepatocytes (MH) isolated from mouse liver, respectively. For the latter, human dermal fibroblasts (HDFs) transduced with GFP for 2.25 days (GFP), or 2.25 days (FHH-2.25d) and 5 days (FHH-5d) after transduction of *FOXA3*, *HNF1A*, and *HNF4A* (FHH). During database search of peptides, proteins were also simultaneously quantified by MaxQuant (v.1.4.1.2)[12] that summed up eXtracted Ion Current (XIC) of all isotopic clusters associated with each mappable protein sequence. Then, the global centering (GC) method[13] was used to normalize proteomic data for each sample, in which the average intensity value of all proteins was normalized to 1 (Mean=1).

From the proteomic data of mouse hepatocyte maturation, we identified 31,318 peptides and 6250 proteins from 6 samples (Supplementary Fig. 12a). The average spectral count of mappable peptides was 2.19, and 15,586 (49.77%) peptides were matched with ≥ 2 spectral counts (Supplementary Fig. 12b). The average number of peptides for mappable proteins was 5.34, and only 1276 (20.42%) proteins were quantified with one matched peptide (Supplementary Fig. 12c). Two-way hierarchical clustering was performed by calculating the Spearman's correlation coefficient for the proteomic data between pairs of samples, and the results indicated that CLiP-Hep and MH cells could be unambiguously distinguished (Supplementary Fig. 12d). From the proteomic data, 16 of 28 predicted central PKs were not detected at all, and

only one newly identified PK, *Prkx*, was significantly up-regulated in CLiP-Hep cells (Supplementary Fig. 12e).

Using the p-sites normalized by their corresponding protein expression levels, we re-analyzed the trans-omic data, and predicted 36 potentially central PKs, in which the three newly identified PKs, *Prkaca*, *Prkacb*, and *Prkx*, were not included (Supplementary Fig. 12f). In addition, we evaluated the accuracy of CKI with or without transcriptomic data. By excluding transcriptomic data, 31 potentially central PKs were predicted merely from phosphoproteomic data, including all the three newly validated PKs (Supplementary Fig. 12g), indicating that including transcriptomic data or not did not influence the final validations.

For human hepatic reprogramming, we identified 28,651 peptides and 5446 proteins from proteomic data (Supplementary Fig. 13a). The average spectral count of mappable peptides was 2.15, and 13,638 (47.60%) peptides were matched with ≥ 2 spectral counts (Supplementary Fig. 13b). The average number of peptides for mappable proteins was 5.52, and only 976 (17.92%) proteins were quantified with one matched peptide (Supplementary Fig. 13c). To evaluate the potential influence of protein expression on the quantification of p-sites, DRPs were identified and compared, using p-sites with or without normalization by their corresponding protein expression levels. From the results, it could be found that including proteomic data or not did not markedly change the phosphoproteomics-based results. For example, 44 DRPs were identified on A-kinase anchor protein 12 (AKAP12) from phosphoproteomic data, and 43 of these p-sites remained to be DRPs after normalization of p-site intensities by proteomic data (Supplementary Fig. 13d). From the proteomic data, only 3 of 15 predicted central PKs, including MAPK4, ROCK2, and *KALRN*, were detected, and only MAPK4 exhibited a > 2-fold change at FHH-5d against FHH-2.25d or GFP (Supplementary Fig. 13e).

Again, we re-analyzed the transcriptomic data and phosphoproteomic data using the p-sites normalized by their corresponding protein expression levels, and predicted 19 potentially central PKs involved in hepatic reprogramming.

Only one validated PK, PIM1, was included (Supplementary Fig. 13f). By excluding transcriptomic data, 13 potentially central PKs including a validated PK, PIM2, were predicted merely from phosphoproteomic data. However, the most important finding of this study, PIM1, could not be covered any longer (Supplementary Fig. 13g).

Our study on human hepatic reprogramming has been started since 2014, and at that time we used the human proteome set from UniProt (Version 201401)[14] for database search. To test whether the update of UniProt will influence the p-site identification, we downloaded the recently released human reference proteome set from UniProt (Version 202203)[14]. It could be found that different versions of reference proteome sets did not significantly change the phosphoproteomic results, and only 305 (5.4%) p-sites identified from UniProt Version 201401 were not covered by UniProt Version 202203 (Supplementary Fig. 13h). Using UniProt Version 202203, we re-analyzed the trans-omic data, and CKI could still recall 13 of 15 previously predicted PKs, including the two validated PKs, PIM1 and PIM2 (Supplementary Fig. 13i).

Supplementary Figures

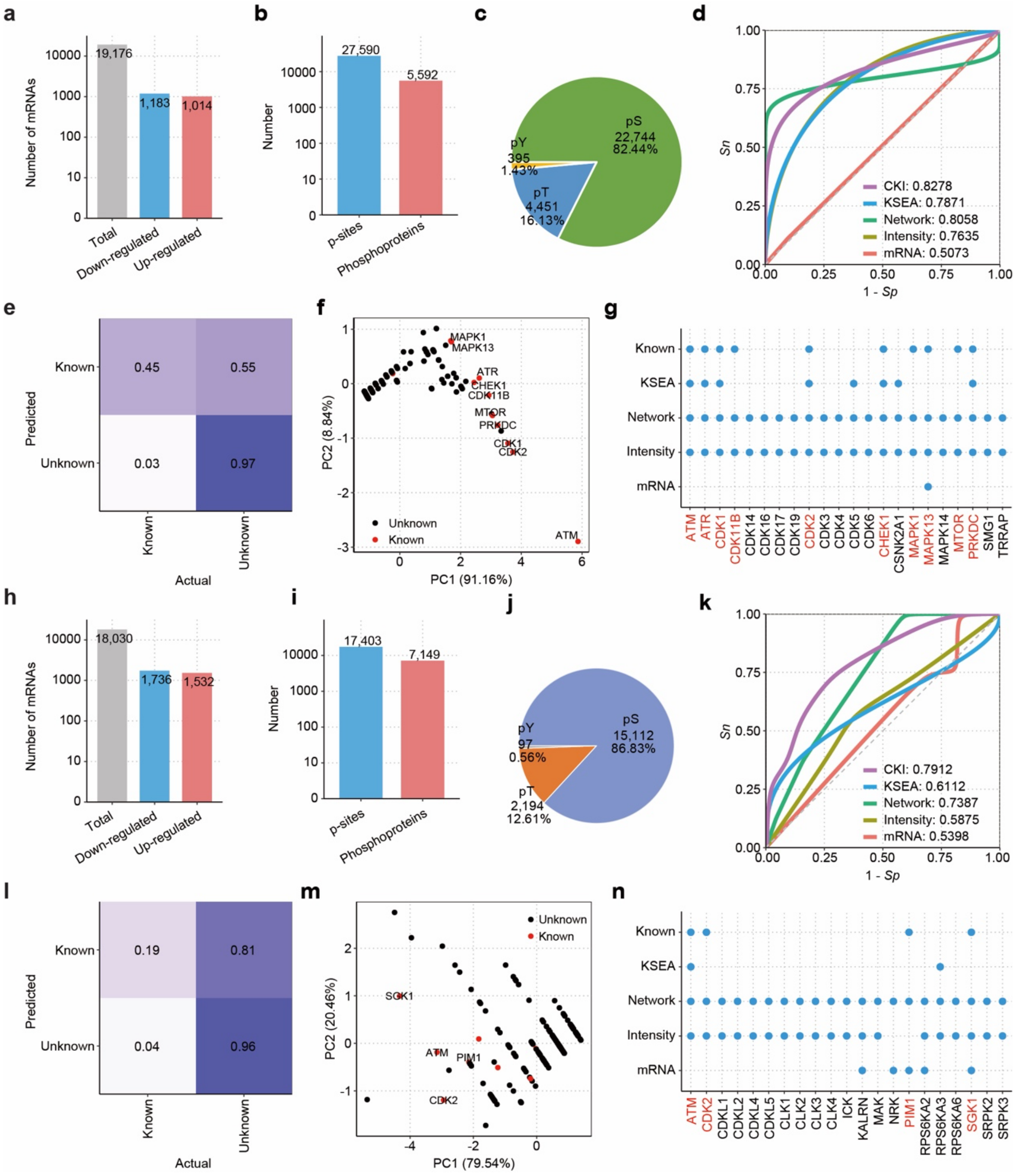

Supplementary Figure 1 | Evaluation of CKI performance on two known datasets. Data were obtained from two previously reported drug-resistance studies: doxorubicin (DOX) resistance of U-2 OS cells (**a-g**) and genistein resistance in breast cancer cells (**h-n**). **a** Number of total mRNAs, up-regulated DEMs, and down-regulated DEMs in samples treated with DOX compared to the control. **b** Total number of quantified p-sites and phosphoproteins. **c** Distribution of phosphorylated residues identified: phosphoserine (pS),

phosphothreonine (pT), and phosphotyrosine (pY) residues. **d** The ROC curves and AUC values for predictions made on the testing set with CKI, KSEA[3,4], and models using each type of data (mRNA expression, substrate p-site intensity, and kinase-substrate network) singly. **e** Confusion matrix for CKI using a threshold of ≥ 14 of 19 pairwise comparisons. **f** Two-dimensional PCA of known and unknown PKs from the CKI predictions. **g** Contribution of each of the three types of data to the final CKI prediction and comparison to KSEA[3,4] predictions. Known PKs reported to be associated with DOX resistance are shown in red. **h** Number of total mRNAs, up-regulated DEMs, and down-regulated DEMs in samples treated with genistein compared to the control. **i** Total number of quantified p-sites and phosphoproteins. **j** Distribution of quantified pS, pT, and pY residues. **k** Comparison of accuracy between CKI and other methods. **l** Confusion matrix of CKI using a threshold of ≥ 6 of 12 pairwise comparisons. **m** PCA of known and unknown PKs from the CKI prediction. **n** Comparison of CKI predictions to other methods. Known PKs reported to be associated with genistein resistance are shown in red.

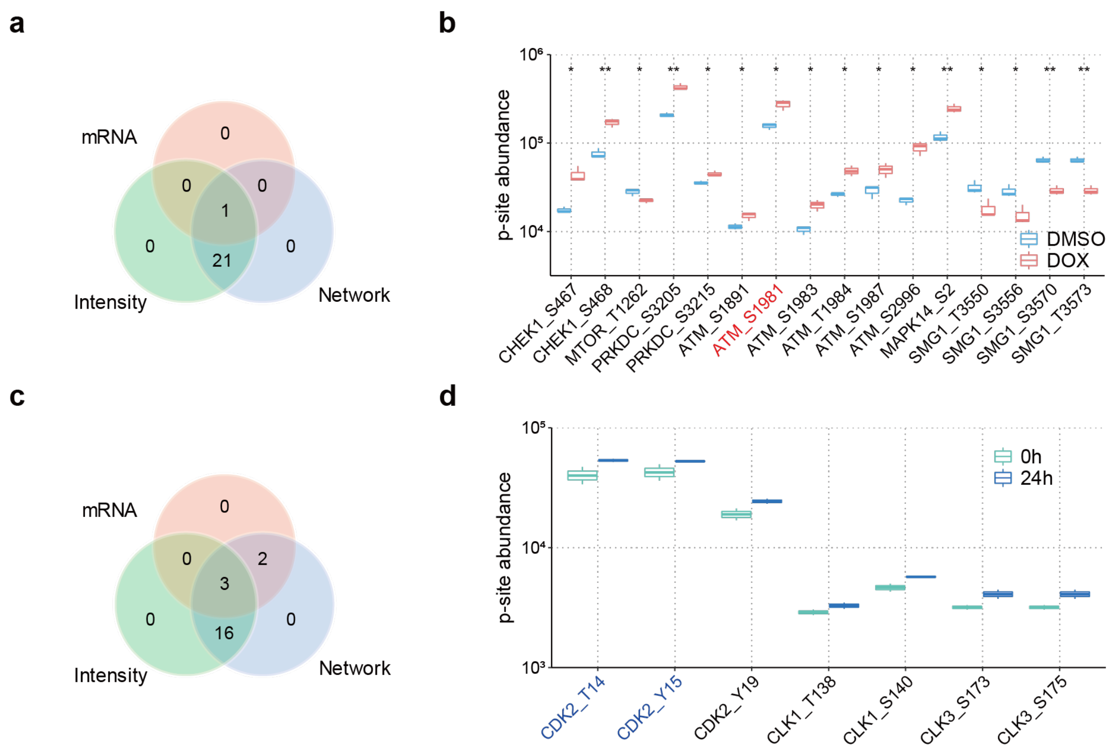

Supplementary Figure 2 | Additional analysis of two drug-resistance studies. a, b For the DOX resistance dataset, the overlap of potentially central PKs derived from different data types (**a**) and p-sites in PKs quantified from phosphoproteomic data if available, including CHEK1 S467 (p = 0.0376), CHEK1 S468 (p = 0.0044), MTOR T1262 (p = 0.0462), PRKDC S3205 (p = 0.0047), PRKDC S3215 (p = 0.0205), ATM S1891 (p = 0.0402), ATM S1981 (p = 0.0208), ATM S1983 (p = 0.0185), ATM T1984 (p = 0.0239), ATM S1987 (p = 0.0398), ATM S2996 (p = 0.0172), MAPK14 S2 (p = 0.0057), SMG1 T3550 (p = 0.0325), SMG1 S3556 (p = 0.0222), SMG1 S3570 (p = 0.0009), and SMG1 T3573 (p = 0.0009). (**b**). n = 3 technical replicates. Box and whisker plots present the means (lines inside the boxes), the 1st and 3rd quartiles (bottom and top bounds of the boxes), and the extents of the data (whiskers). **c, d** For the genistein resistance dataset, the overlap of potentially central PKs derived from different data types (**c**) and quantified p-sites in PKs (**d**). n = 2 biological replicates. Box and whisker plots present the means (lines inside the boxes), the 1st and 3rd quartiles (bottom and top bounds of the boxes), and the extents of the data (whiskers). The PK activity-associated p-site(s) are shown in red

(positively associated with PK activity) or blue (negatively associated with the PK activity).

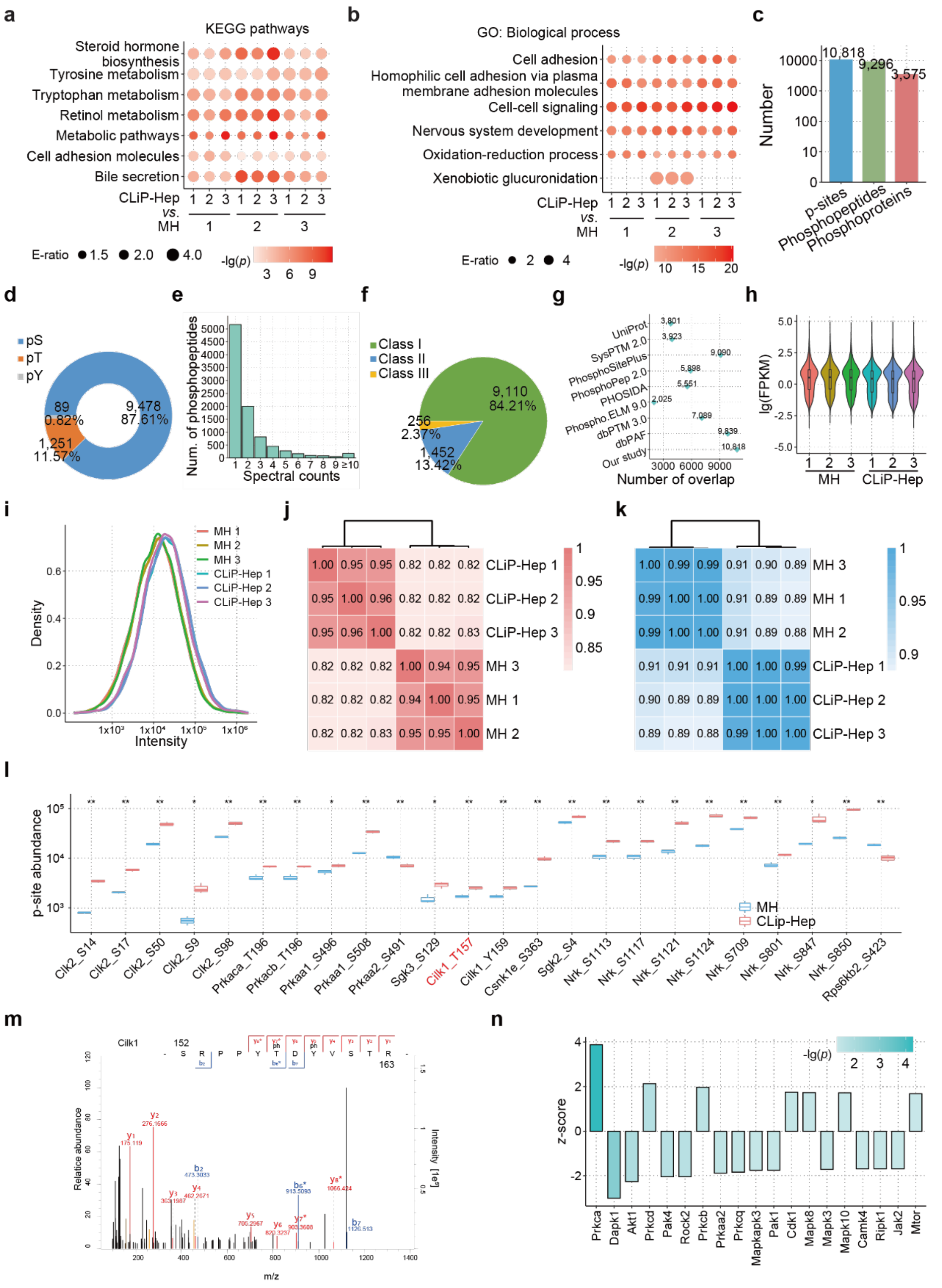

Supplementary Figure 3 | Additional analyses of the trans-omic data for mouse hepatocyte maturation. a, b KEGG- (**a**) and GO-based (**b**) enrichment analyses of DEMs in CLiP-Hep *vs.* MH. **c** Number of quantified p-sites, phosphopeptides, and phosphoproteins. **d** Distribution of quantified pS, pT, and pY residues. **e** Distribution of phosphopeptides with different numbers of

spectral counts. **f** Classification of all quantified p-sites based on localization probability (LP) scores. **g** Comparison of quantified p-sites with known p-sites integrated from eight public databases. **h, i** Distribution of FPKM values for mapped mRNAs (**h**) or TMT intensities for p-sites (**i**) in each sample. $n = 3$ biological replicates. Box and whisker plots present the means (lines inside the boxes), the 1st and 3rd quartiles (bottom and top bounds of the boxes), and the extents of the data (whiskers). **j, k** Two-way hierarchical clustering of the three biological replicates of MH and CLiP-Hep samples using transcriptomes (**j**) or phosphoproteomes (**k**). **l** Quantified p-sites in 28 potentially central PKs (if available), including Clk2 S14 ($p = 0.0023$), Clk2 S17 ($p = 0.0029$), Clk2 S50 ($p = 0.0046$), Clk2 S9 ($p = 0.0267$), Clk2 S98 ($p = 0.0072$), Prkaca T196 ($p = 0.0054$), Prkacb T196 ($p = 0.0054$), Prkaa1 S496 ($p = 0.0150$), Prkaa1 S508 ($p = 0.0022$), Prkaa2 S491 ($p = 0.0050$), Sgk3 S129 ($p = 0.0118$), Cilk1 T157 ($p = 0.0052$), Cilk1 Y159 ($p = 0.0052$), Csnk1e S363 ($p = 0.0059$), Sgk2 S4 ($p = 0.0095$), Nrk S1113 ($p = 0.0005$), Nrk S1117 ($p = 0.0005$), Nrk S1121 ($p = 0.0011$), Nrk S1124 ($p = 0.0025$), Nrk S709 ($p = 0.0041$), Nrk S801 ($p = 0.0029$), Nrk S847 ($p = 0.0379$), Nrk S850 ($p = 0.0001$), and Rps6kb2 S423 ($p = 0.0049$). The p-site Cilk1 T157, which is positively associated with PK activity, is shown in red. *$p < 0.05$, **$p < 0.01$ (2-sided Student's *t*-test). **m** The MaxQuant MS/MS spectrum of the phosphopeptide for T157 and Y159 in Cilk1. **n** Potentially central PKs predicted by KSEA[3,4] ($p < 0.05$).

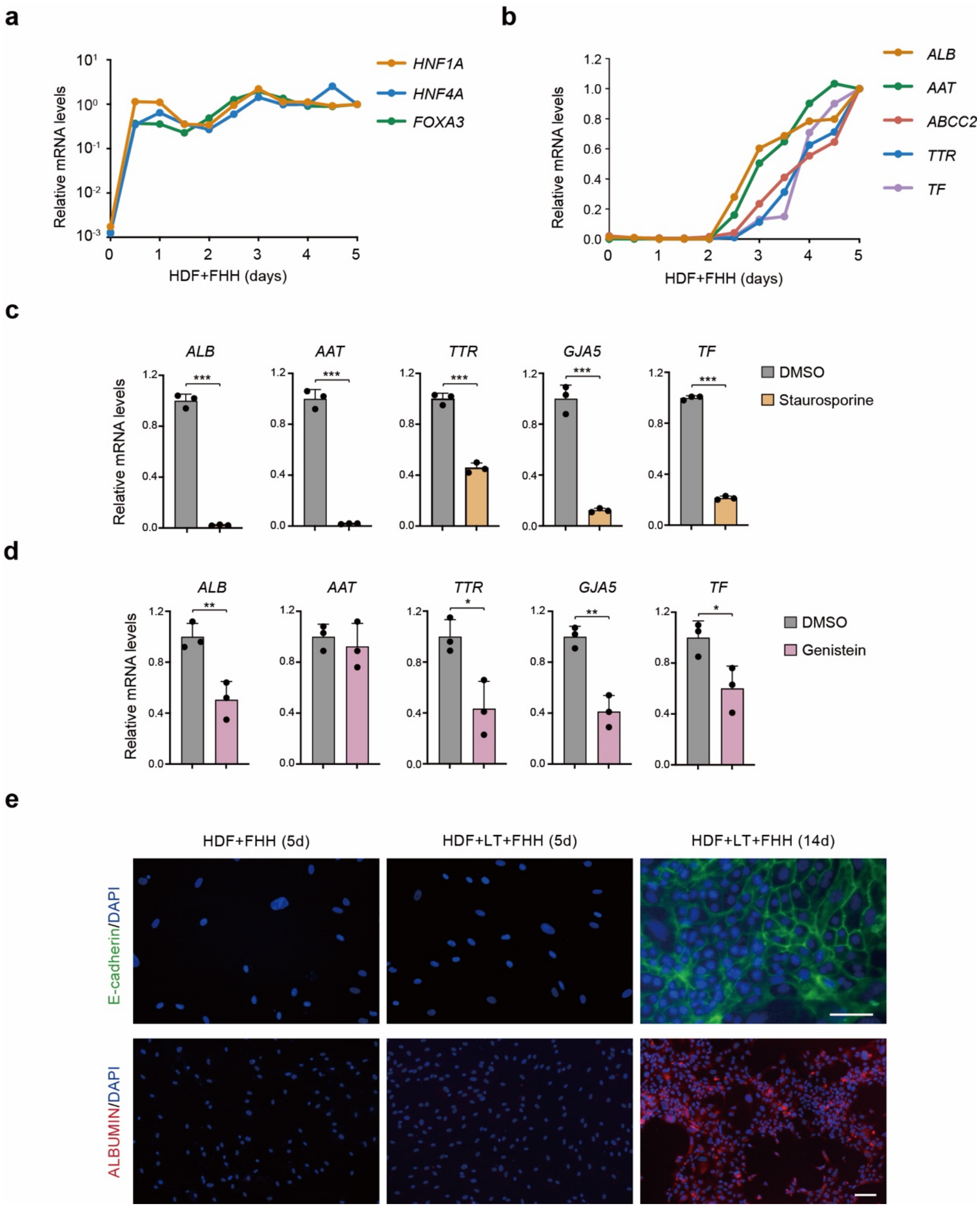

Supplementary Figure 4 | PKs are important for hepatic reprogramming. **a, b** Expression of FHH (**a**) and hepatic genes (**b**) were quantified at different time points after transduction of FHH. **c, d** Treatment with the protein kinase inhibitors staurosporine (**c**) and genistein (**d**) suppressed induction of liver genes in HDFs transfected with FHH for 5 days (Staurosporine treatment: *ALB* $p < 0.0001$, *AAT* $p < 0.0001$, *TTR* $p < 0.0001$, *GJA5* $p = 0.0002$, *TF* $p < 0.0001$; Genistein treatment: *ALB* $p = 0.0088$, *TTR* $p = 0.0182$, *GJA5* $p = 0.0024$, *TF* $p = 0.00349$; $n = 3$). (**e**) Immunofluorescence staining of ALBUMIN and E-

cadherin in HDFs transfected with FHH or FHH+LT (SV40 large T) for indicated days. Scale bars = 100 μm. *n* = 2 biological replicates. Data are shown as the mean + standard deviation. *$p < 0.05$, **$p < 0.01$, ***$p < 0.001$ (unpaired two-sided Student's *t*-test). Source data are provided as a Source Data file.

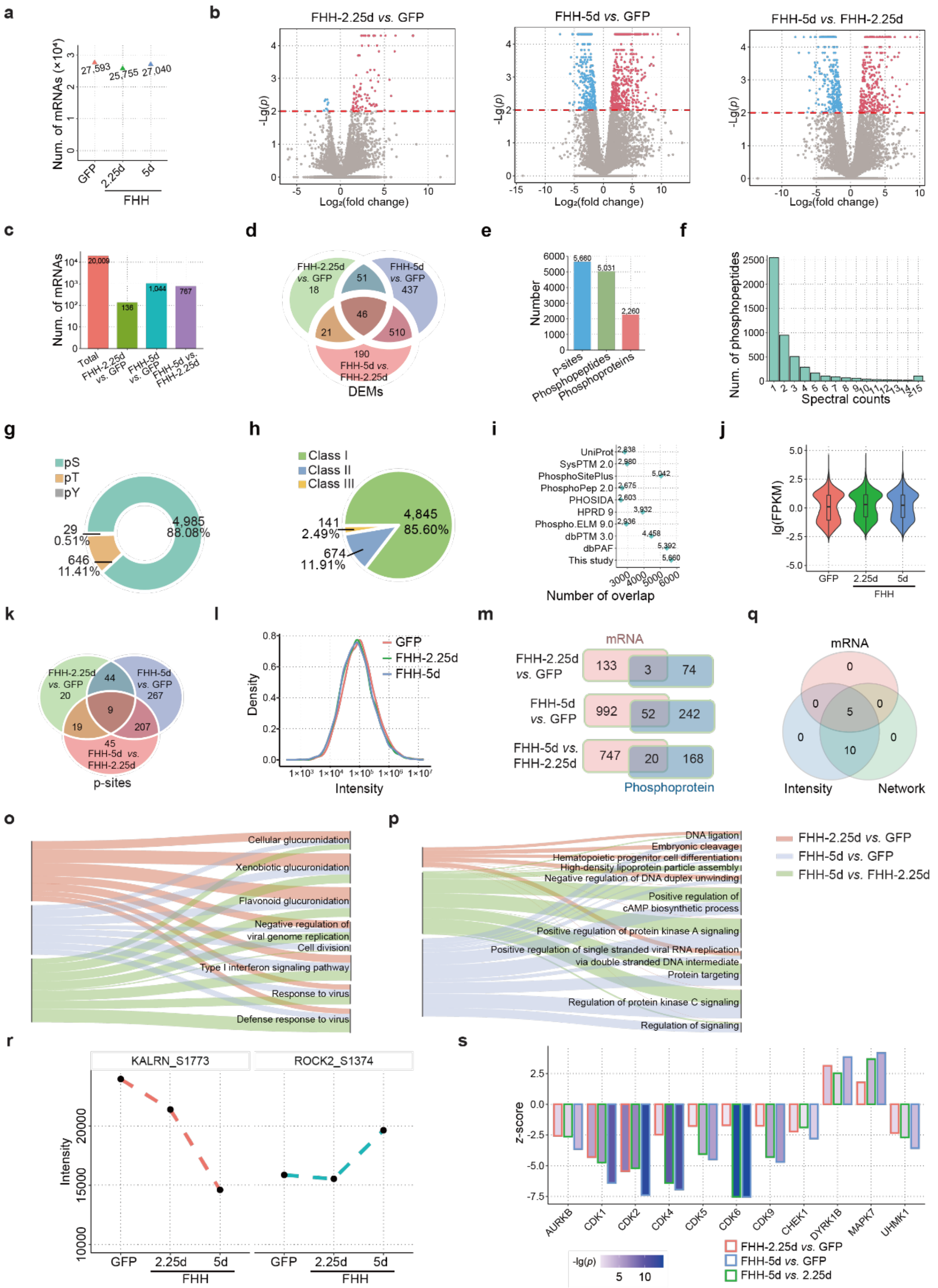

Supplementary Figure 5 | Additional analyses of the trans-omic data for HDFs undergoing hepatic conversion. a Number of mRNAs mapped with at least one clean read in the indicated samples. **b** Volcano plot showing differential expression in FHH-2.25d *vs*. GFP, FHH-5d *vs*. GFP, and FHH-5d *vs*. FHH-2.25d. Statistical tests were implemented in Cuffdiff program with default

parameters. No adjustments were made for multiple comparisons. Red or blue dots indicate the genes upregulated in former or latter conditions. **c** Number of total mRNAs and DEMs from all pairwise sample comparisons. **d** Venn diagram showing the number of DEMs in the indicated comparisons. **e** Number of p-sites, phosphopeptides, and phosphoproteins. **f** Distribution of phosphopeptides with different numbers of spectral counts. **g** Distribution of quantified pS, pT, and pY residues. **h** Classification of all quantified p-sites based on LP scores. **i** Comparison of p-sites quantified in this study to known p-sites published in nine public databases. **j** Distribution of FPKM values for mapped mRNAs in each sample. **k** Venn diagram showing the number of DRPs in the indicated comparisons. **l** Intensity distribution of p-sites quantified in the three samples. **m** Overlap of DEMs from transcriptomic data and proteins containing at least one DRP from phosphoproteomic data. **o, p** GO-based enrichment analyses of DEMs (**o**) and DRPs (**p**). **q** Number of potentially central PKs predicted from different data types from all pairwise comparisons. **r** Two quantified p-sites in potentially central PKs (S1773 of KALRN and S1374 of ROCK2) identified from the phosphoproteomic data. **s** Potentially central PKs predicted by KSEA[3,4] ($p < 0.05$).

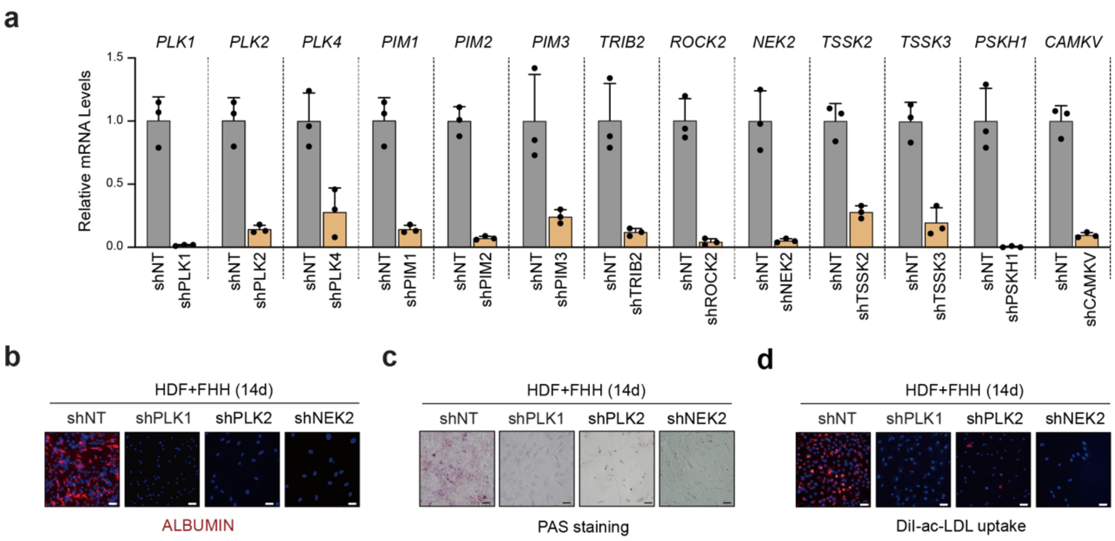

Supplementary Figure 6 | Validation of PKs required for efficient hepatic reprogramming. a Knockdown efficiencies of shRNAs targeting each of the candidate PKs as quantified by qRT-PCR (n = 3). Data are shown as the mean + standard deviation. **b-d** Representative images of ALBUMIN immunofluorescence staining (**b**), PAS staining (**c**) and DiI-ac-LDL uptake assay (**d**) of hepatic reprogramming samples upon knockdown of indicated PK genes. Scale bars = 100 μm. n = 2 biological replicates. Source data are provided as a Source Data file.

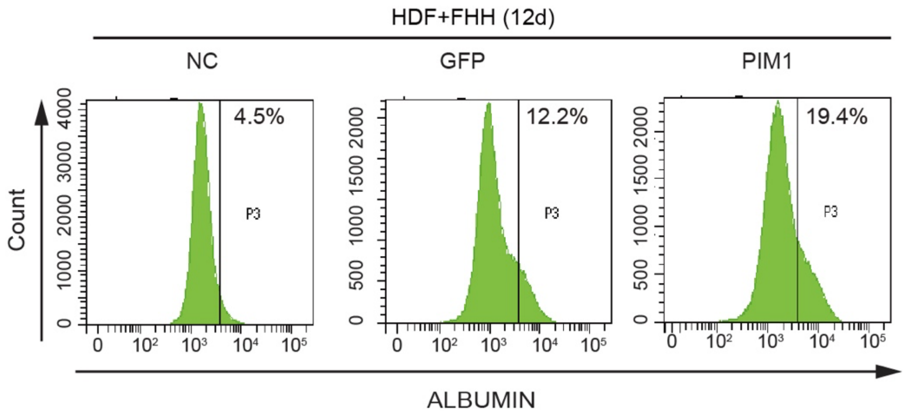

Supplementary Figure 7 | Role of PIM1 in the increase of ALUBMIN positive cells during hepatic reprogramming. Representative flow plots for ALBUMIN immunofluorescence staining in *GFP*- or *PIM1*-overexpressing HDFs after transduction of FHH for 12 days.

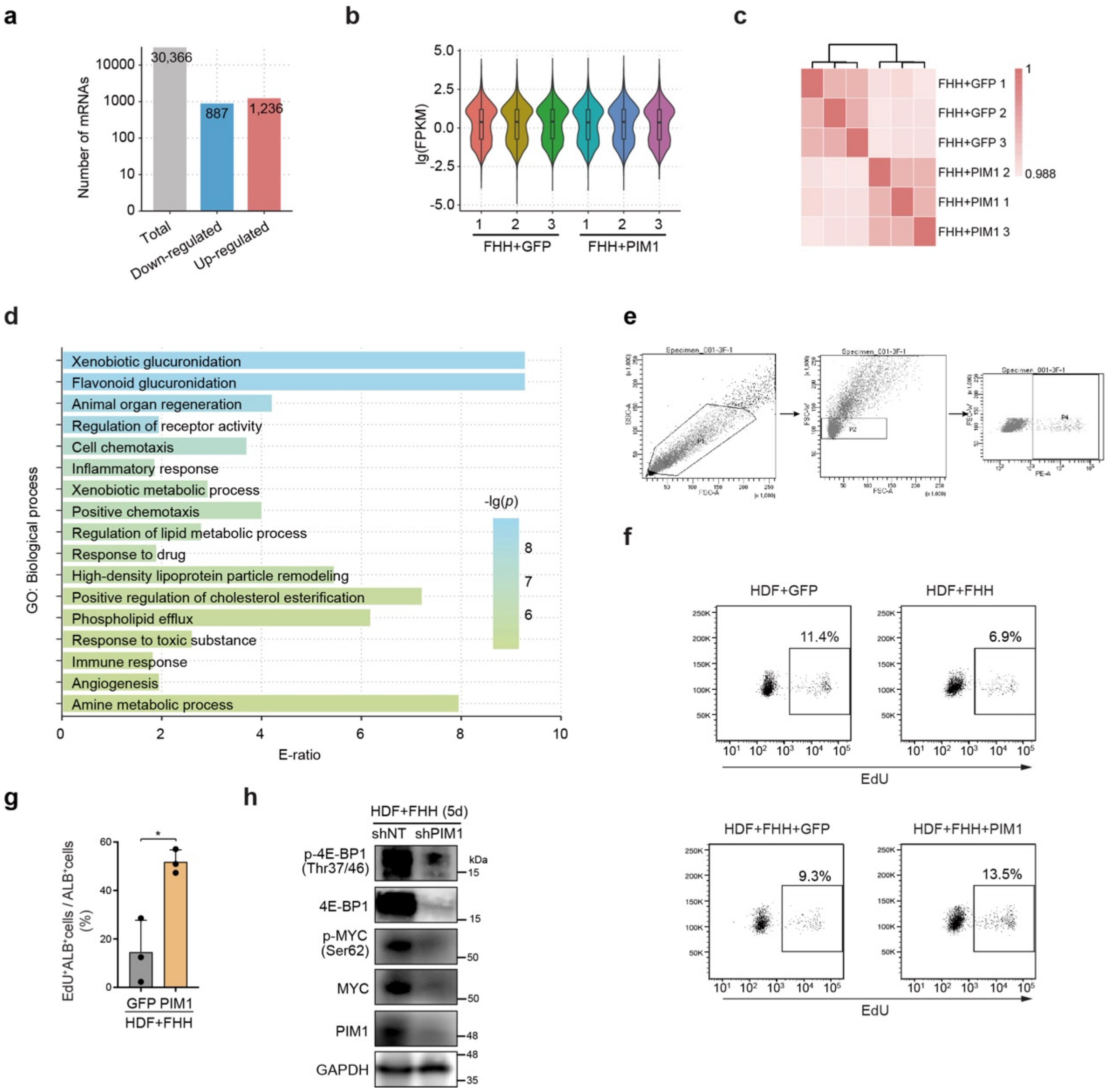

Supplementary Figure 8 | Regulation of cell cycle by PIM1. a Number of total mRNAs and DEMs from HDF+FHH+PIM1 compared to HDF+FHH+GFP. **b** Distribution of FPKM values of mapped mRNAs in each sample. **c** Two-way hierarchical clustering of transcriptomic data for the three biological replicates of FHH+PIM1 or FHH+GFP. **d** GO-based enrichment analysis of biological processes for DEMs in FHH+PIM1 *vs*. FHH+GFP samples. **e** Gating strategy for flow cytometry analysis shown in Fig. 5f, 5h, Supplementary Fig. 7 and 8f. Cells were gated based on size and granularity using FSC-A vs SSC-A to eliminate debris and clumped cells. **f** Representative flow plots for EdU staining assay in HDFs overexpressing indicated genes for 12 days. **g** Percentage of EdU+ cells among ALBUMIN+ cells (p = 0.0103, n = 3). Data are shown as the

mean + standard deviation. $*p < 0.05$ (unpaired two-sided Student's *t*-test). **h** Immunoblotting of PIM1 downstream substrate proteins in HDFs infected with FHH and PIM1/non-targeted shRNAs for 5 days. $n = 2$ biological replicates. Data are shown as the mean ± standard deviation. $*p < 0.05$ (unpaired two-sided Student's *t*-test). Source data are provided as a Source Data file.

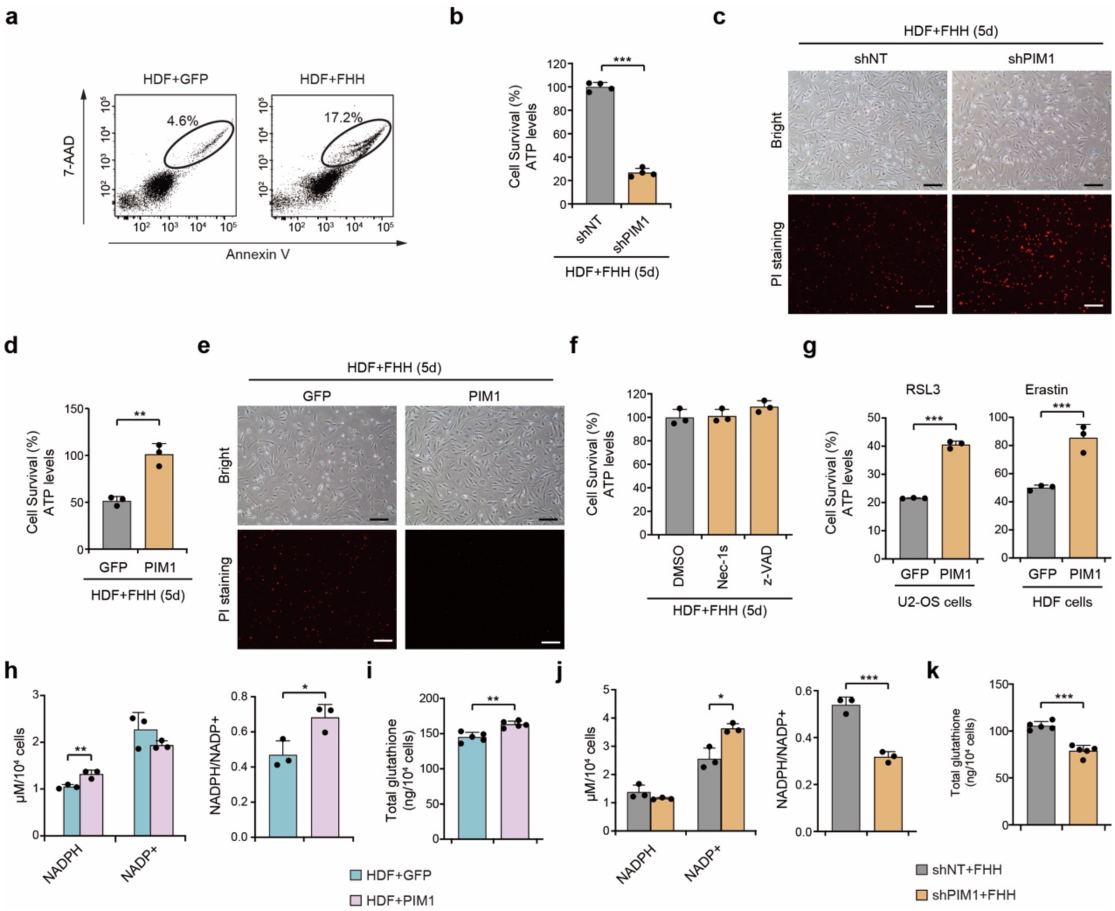

Supplementary Figure 9 | Regulation of ferroptosis by PIM1. a Representative flow plots for Annexin V/7-AAD staining showing FHH-induced cell death on day 5. **b** Quantification of cell viability of HDFs after transduction of FHH with non-targeting or *PIM1* shRNAs for 5 days using ATP assay (p < 0.0001, n = 4). **c** Propidium iodide (PI) staining assay showing the increased cell death of HDFs transduced with FHH and *PIM1* shRNA for 5 days. Scale bars = 100 μm. n = 4 biological replicates. **d** Quantification of cell viability of HDFs after transduction of FHH with *GFP* or *PIM1* for 5 days using ATP assay (p = 0.0024, n = 3). **e** Propidium iodide (PI) staining assay showing the reduced cell death of HDFs transduced with FHH and *PIM1* for 5 days. Scale bars = 100 μm. n = 4 biological replicates. **f** Quantification of cell viability of HDF+FHH treated with DMSO, Nec-1s or z-VAD (n = 3). **g** Quantification of cell viability of U2-OS cells overexpressing *GFP* or *PIM1* treated with RSL3 (p < 0.0001), and HDF cells overexpressing *GFP* or *PIM1* treated with Erastin (p = 0.0033). **h** Cellular NADP/NADPH levels on day 5 of HDFs transduced with *GFP* or *PIM1*

(NADPH *p* = 0.0088, NADPH/NDP+ *p* = 0.0263, n = 3). **i** Cellular GSH levels on day 5 of HDFs transduced with *GFP* or *PIM1* overexpression (*p* = 0.0011, *n* = 3). **j** Cellular NADP/NADPH levels on day 5 of hepatic transdifferentiation with *GFP* or *PIM1* shRNA overexpression (NADP+ *p* = 0.0111, NADPH/NDP+ *p* = 0.0008, *n* = 3). **k** Cellular GSH levels on day 5 of hepatic transdifferentiation with *GFP* or *PIM1* shRNA overexpression (*p* < 0.0001, *n* = 3). Data are shown as the mean ± standard deviation. **p* < 0.05, ***p* < 0.01, ****p* < 0.001 (unpaired two-sided Student's *t*-test). Source data are provided as a Source Data file.

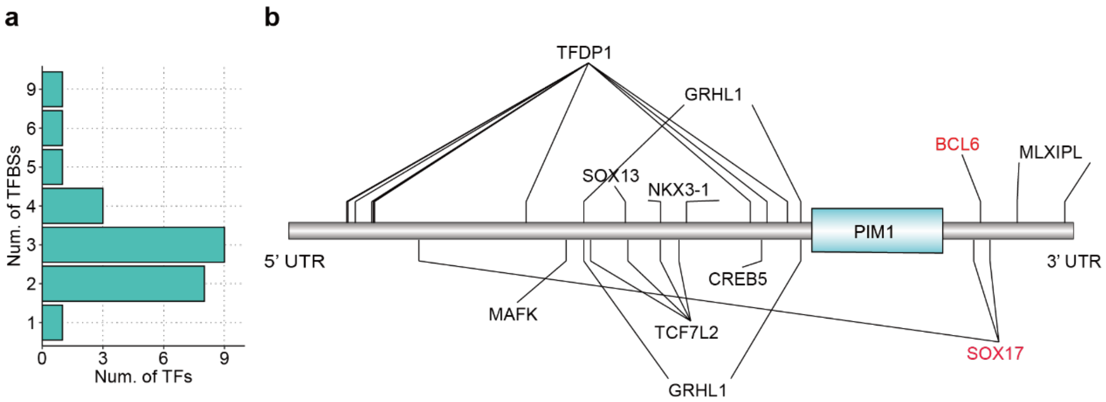

Supplementary Figure 10 | Additional analysis of the TPCW. a Distribution of TFBSs in the upstream or downstream regions of the 24 FHH-regulated TFs. **b** TFBSs regulated by 10 FHH-regulated TFs in the upstream or downstream region of PIM1. It has been reported that PIM1 was also transcriptionally regulated by an additional TF, STAT4, but the exact TFBSs were not available[15].

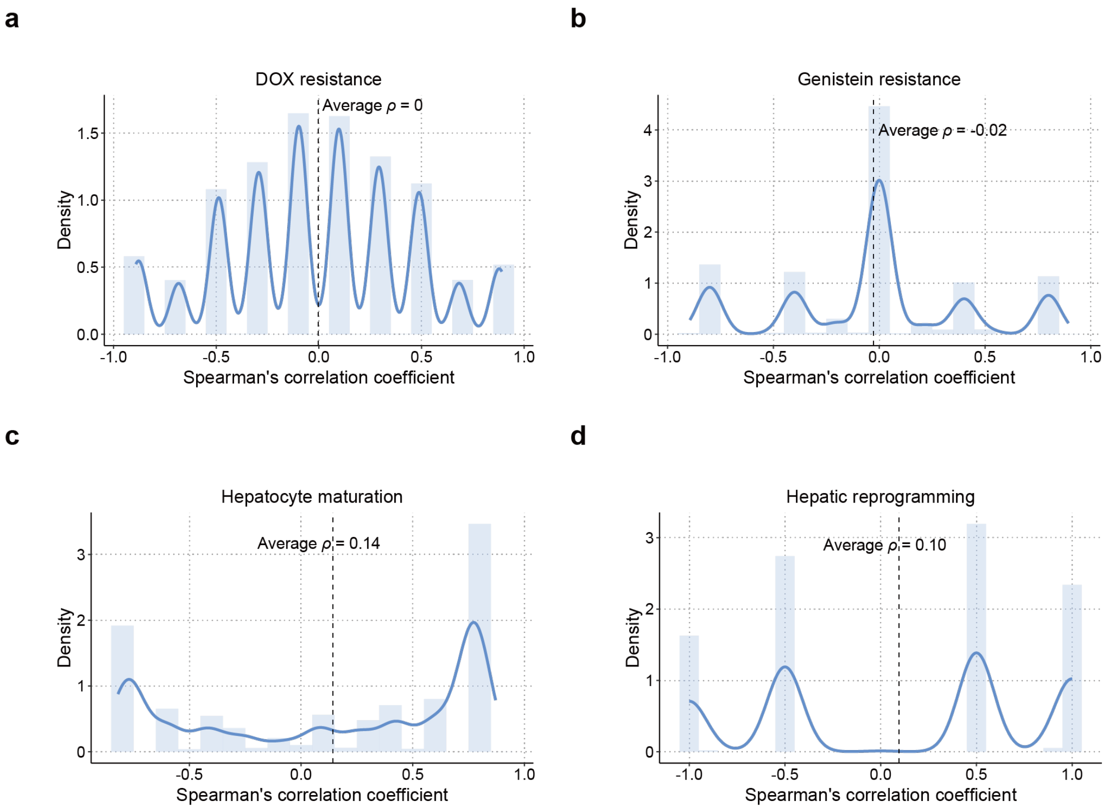

Supplementary Figure 11 | Correlations between mRNA expression levels of PKs and the corresponding substrate p-site intensities. a-d Based on the predicted ssKSRs for each process, the average Spearman's correlation coefficients for mRNA expression of PKs and the corresponding substrate p-site intensities were calculated for DOX resistance (**a**), genistein resistance (**b**), mouse hepatocyte maturation (**c**), and hepatic programming (**d**).

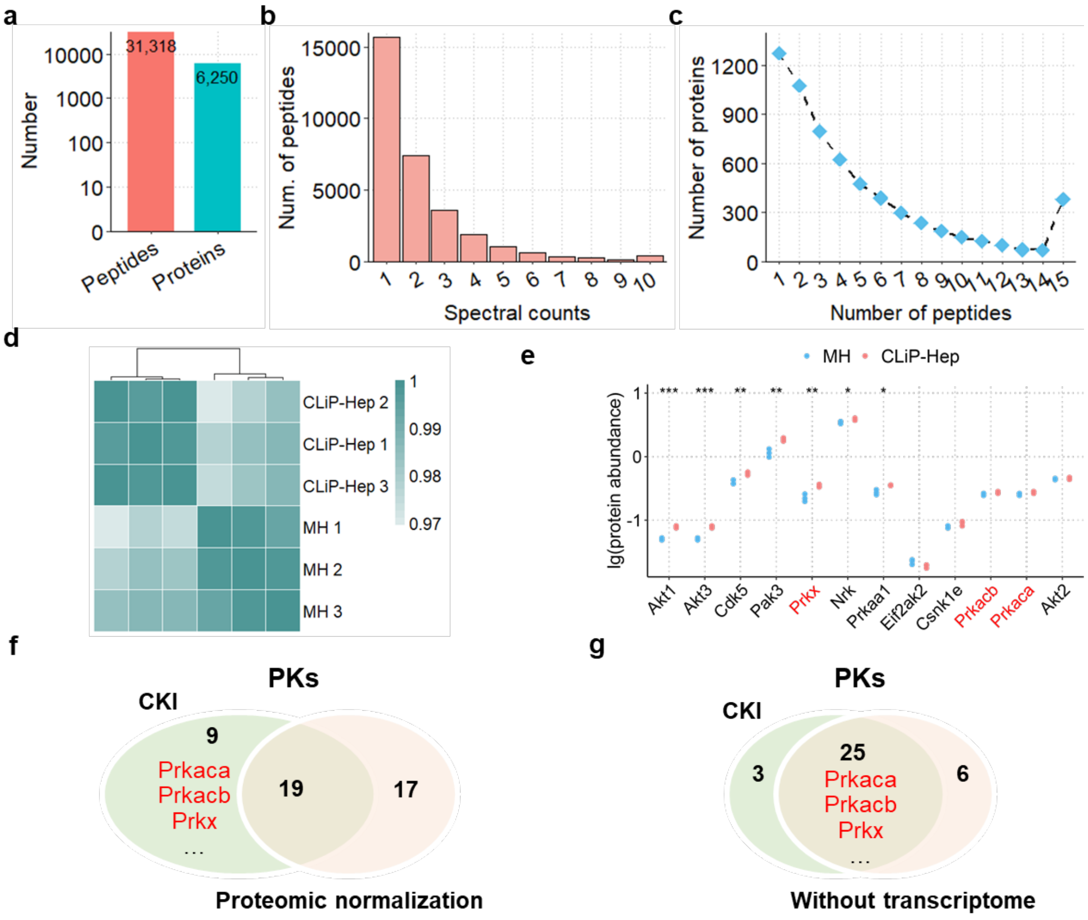

Supplementary Figure 12 | Analyses of proteomic data for mouse hepatocyte maturation. a Number of identified peptides and proteins. **b** Distribution of peptides with different numbers of spectral counts. **c** Distribution of proteins with different numbers of identified peptides. **d** Two-way hierarchical clustering of the three biological replicates of MH and CLiP-Hep samples using proteomic data. **e** Normalized quantification levels of 12 detected PKs that were included in the 28 predicted central PKs, including Akt1 (p = 0.0005), Akt3 (p = 0.0005), Cdk5 (p = 0.0046), Pak3 (p = 0.0058), Prkx (p = 0.0078), Nrk (p = 0.0224), and Prkaa1 (p = 0.0286). The three validated PKs, including *Prkx*, *Prkacb,* and *Prkaca*, which promote hepatocyte maturation, are shown in red. *$p < 0.05$, **$p < 0.01$, ***$p < 0.001$ (2-sided Student's t-test). **f** The overlap of CKI predictions, using p-sites with or without normalization by their corresponding protein expression levels. **g** The overlap of CKI predictions, using transcriptomic data or not.

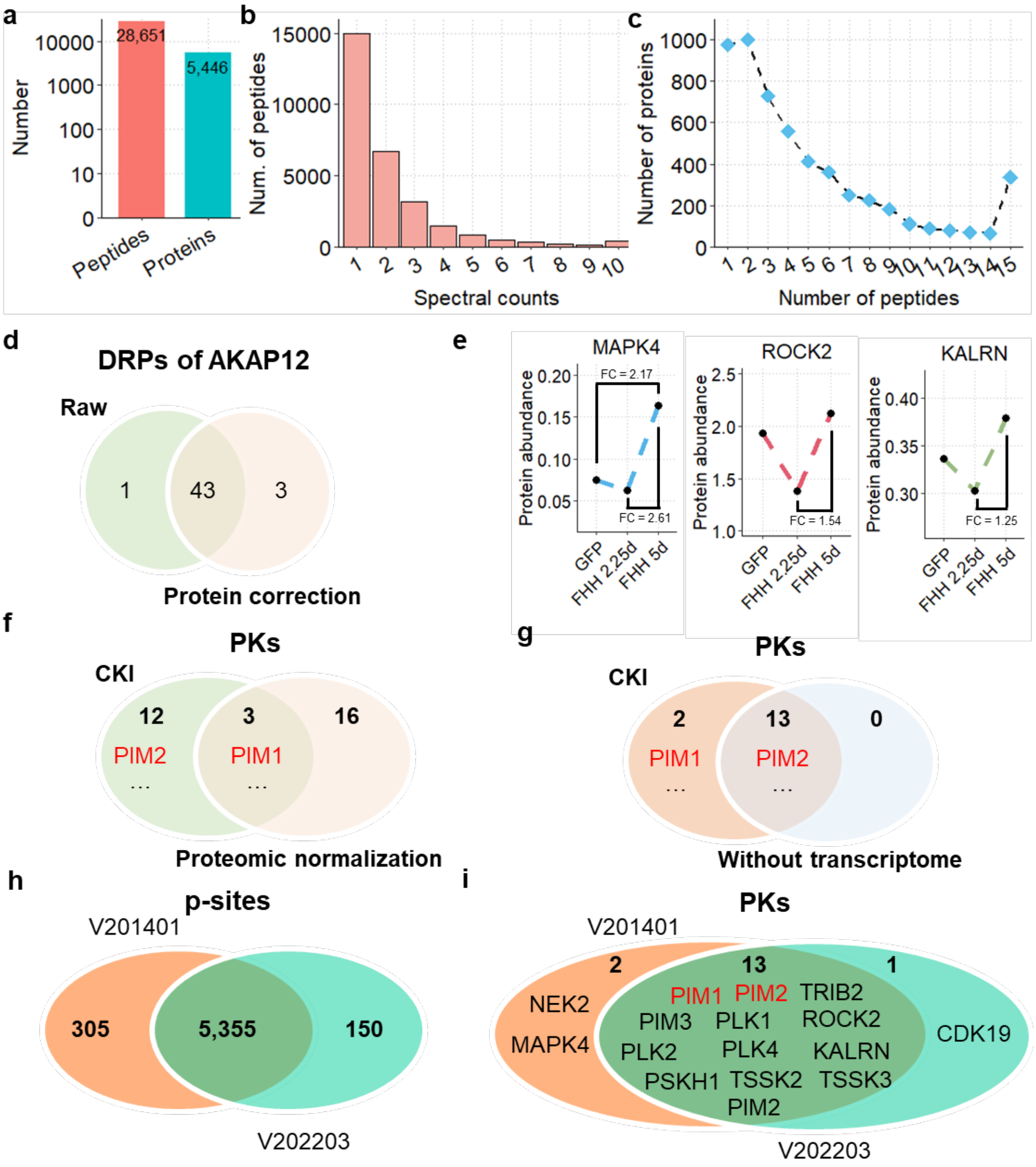

Supplementary Figure 13 | Analyses of proteomic data for human hepatic reprogramming. **a** Number of identified peptides and proteins. **b** Distribution of peptides with different numbers of spectral counts. **c** Distribution of proteins with different numbers of identified peptides. **d**. The overlap of identified DRPs in AKAP12, using p-sites with or without normalization by their corresponding protein expression levels. **e** Normalized quantification levels of 3 detected PKs that were included in the 15 predicted central PKs. FC, fold change. **f** The overlap of CKI predictions, using p-sites with or without normalization by their corresponding protein expression levels. **g** The overlap of CKI predictions, using transcriptomic data or not. **h** The overlap of p-sites identified using

different versions of the human reference proteome sets. **i** The overlap of PKs predicted by CKI, using different versions of the human reference proteome sets.

Supplementary References

1. Zanotto-Filho A*, et al.* Alkylating Agent-Induced NRF2 Blocks Endoplasmic Reticulum Stress-Mediated Apoptosis via Control of Glutathione Pools and Protein Thiol Homeostasis. *Mol. Cancer Ther.* **15**, 3000-3014 (2016).
2. Hogrebe A, von Stechow L, Bekker-Jensen DB, Weinert BT, Kelstrup CD, Olsen JV. Benchmarking common quantification strategies for large-scale phosphoproteomics. *Nat. Commun.* **9**, 1045 (2018).
3. Casado P*, et al.* Kinase-substrate enrichment analysis provides insights into the heterogeneity of signaling pathway activation in leukemia cells. *Sci. Signal.* **6**, rs6 (2013).
4. Wiredja DD, Koyutürk M, Chance MR. The KSEA App: a web-based tool for kinase activity inference from quantitative phosphoproteomics. *Bioinformatics* **33**, 3489-3491 (2017).
5. Booth L, West C, Hoff DV, Dent P. GZ17-6.02 and Doxorubicin Interact to Kill Sarcoma Cells via Autophagy and Death Receptor Signaling. *Front. Oncol.* **10**, 1331 (2020).
6. George SA*, et al.* p38δ genetic ablation protects female mice from anthracycline cardiotoxicity. *Am. J. Physiol. Heart Circ. Physiol.* **319**, H775-h786 (2020).
7. Stefanski CD, Keffler K, McClintock S, Milac L, Prosperi JR. APC loss affects DNA damage repair causing doxorubicin resistance in breast cancer cells. *Neoplasia* **21**, 1143-1150 (2019).
8. Lavin MF, Kozlov S. ATM activation and DNA damage response. *Cell Cycle* **6**, 931-942 (2007).
9. Gong P*, et al.* Transcriptomic analysis identifies gene networks regulated by estrogen receptor α (ERα) and ERβ that control distinct effects of different botanical estrogens. *Nucl. Recept. Signal.* **12**, e001 (2014).
10. Fang Y*, et al.* Quantitative phosphoproteomics reveals genistein as a modulator of cell cycle and DNA damage response pathways in triple-negative breast cancer cells. *Int. J. Oncol.* **48**, 1016-1028 (2016).
11. Huang P*, et al.* Direct reprogramming of human fibroblasts to functional and expandable hepatocytes. *Cell Stem Cell* **14**, 370-384 (2014).
12. Tyanova S, Temu T, Cox J. The MaxQuant computational platform for mass spectrometry-based shotgun proteomics. *Nat. Protoc.* **11**, 2301-2319 (2016).
13. Wang C*, et al.* Integrated omics in Drosophila uncover a circadian kinome. *Nat. Commun.* **11**, 2710 (2020).
14. The UniProt Consortium. UniProt: a worldwide hub of protein knowledge. *Nucleic Acids Res.* **47**, D506-d515 (2019).
15. Matikainen S, Sareneva T, Ronni T, Lehtonen A, Koskinen PJ, Julkunen

I. Interferon-alpha activates multiple STAT proteins and upregulates proliferation-associated IL-2Ralpha, c-myc, and pim-1 genes in human T cells. *Blood* **93**, 1980-1991 (1999).
