# Supplementary material for: PIM1 promotes hepatic conversion by suppressing reprogramming-induced ferroptosis and cell cycle arrest
Source: Nat Commun. 2022 Sep 6;13:5237. doi: 10.1038/s41467-022-32976-9 (PMC9448736; doi:10.1038/s41467-022-32976-9)
Supplement: Supplementary file 3 — Description of Additional Supplementary Files [file 41467_2022_32976_MOESM3_ESM.docx]

**Description of Additional Supplementary Files**

**Supplementary Data 1:** The summary of samples and potentially central PKs predicted by CKI with TMT intensities of their corresponding p-sites, for two drug-resistance studies, including DOX resistance in U-2 OS cells and genistein resistance in breast cancer cells, respectively.

**Supplementary Data 2:** The transcriptomic and phosphoproteomic profiling, as well as 28 central PKs predicted by CKI for hepatocyte maturation process of mouse liver progenitor cells.

**Supplementary Data 3:** The transcriptomic and phosphoproteomic profiling, DEMs, and DRPs of hepatic lineage reprogramming.

**Supplementary Data 4:** The details of 15 central PKs predicted with CKI to be potentially involved in regulating hepatic lineage reprogramming, including the p values calculated from the 9 pairwise comparisons, and false positive rate (FPR) values determined from permutation tests.

**Supplementary Data 5:** The transcriptomes of HDFs undergoing hepatic transdifferentiation in the context of PIM1 or GFP overexpression.

**Supplementary Data 6:** The trans-omic modeling of the hepatic transdifferentiation-associated TPCW, including all curated genes of hepatic lineage, ferroptosis, and cell cycle, the 24 TFs potentially regulated by FHH, the TF-target relations, and the ssKSRs.

**Supplementary Data 7:** Clone primers, shRNA oligos, and qRT-PCR primers in this study.
