# Supplementary material for: PIM1 promotes hepatic conversion by suppressing reprogramming-induced ferroptosis and cell cycle arrest
Source: Nat Commun. 2022 Sep 6;13:5237. doi: 10.1038/s41467-022-32976-9 (PMC9448736; doi:10.1038/s41467-022-32976-9)
Supplement: Supplementary file 11 — Reporting Summary [file 41467_2022_32976_MOESM11_ESM.pdf]

Corresponding author(s): Yilai Shu, Yu Xue & Pengyu Huang

Last updated by author(s): Aug 10, 2022

Reporting Summary

Nature Portfolio wishes to improve the reproducibility of the work that we publish. This form provides structure for consistency and transparency in reporting. For further information on Nature Portfolio policies, see our Editorial Policies and the Editorial Policy Checklist.

Statistics

For all statistical analyses, confirm that the following items are present in the figure legend, table legend, main text, or Methods section.

n/a	Confirmed	
☐	☒	The exact sample size (*n*) for each experimental group/condition, given as a discrete number and unit of measurement
☐	☒	A statement on whether measurements were taken from distinct samples or whether the same sample was measured repeatedly
☐	☒	The statistical test(s) used AND whether they are one- or two-sided<br>*Only common tests should be described solely by name; describe more complex techniques in the Methods section.*
☒	☐	A description of all covariates tested
☐	☒	A description of any assumptions or corrections, such as tests of normality and adjustment for multiple comparisons
☐	☒	A full description of the statistical parameters including central tendency (e.g. means) or other basic estimates (e.g. regression coefficient) AND variation (e.g. standard deviation) or associated estimates of uncertainty (e.g. confidence intervals)
☐	☒	For null hypothesis testing, the test statistic (e.g. *F*, *t*, *r*) with confidence intervals, effect sizes, degrees of freedom and *P* value noted<br>*Give P values as exact values whenever suitable.*
☒	☐	For Bayesian analysis, information on the choice of priors and Markov chain Monte Carlo settings
☒	☐	For hierarchical and complex designs, identification of the appropriate level for tests and full reporting of outcomes
☐	☒	Estimates of effect sizes (e.g. Cohen's *d*, Pearson's *r*), indicating how they were calculated

Our web collection on statistics for biologists contains articles on many of the points above.

Software and code

Policy information about availability of computer code

Data collection	For RNA-Seq data, sequencing were performed on an Illumina Hiseq 3000.<br>For phosphoproteomic data, Q ExactiveTM Plus (Thermo) was used to MS/MS analysis.
Data analysis	The R package edgeR (version 3.28.1) was used to calculate differentially expressed mRNAs.<br>MaxQuant (v.1.4.1.2) was used for standard database search of MS/MS raw data.<br>The transcriptomic analysis was produced with Bowtie 2 (version 2.2.4), TopHat (version 2.2.1), and Cufflinks (version 2.2.1).<br>iGPS was used to predict the upstream regulatory PKs of identified p-sites.<br>For the human and mouse phosphoproteomic data, the R package KSEAapp (version 0.99.0) was used to identify potentially central PKs (p < 0.05).<br>Network was constructed and visualized with Cytoscape 3.8.2 software.<br>The source code of CKI has been uploaded to GitHub (https://github.com/BioCUCKOO/CKI).

For manuscripts utilizing custom algorithms or software that are central to the research but not yet described in published literature, software must be made available to editors and reviewers. We strongly encourage code deposition in a community repository (e.g. GitHub). See the Nature Portfolio guidelines for submitting code & software for further information.

Data

Policy information about availability of data

All manuscripts must include a data availability statement. This statement should provide the following information, where applicable:

- Accession codes, unique identifiers, or web links for publicly available datasets
- A description of any restrictions on data availability
- For clinical datasets or third party data, please ensure that the statement adheres to our policy

The read count files of U-2 OS cells and genistein resistance were downloaded from Gene Expression Omnibus (GEO, https://www.ncbi.nlm.nih.gov/geo/, accession number: GSE84863, GSE56066).
The searched results of TMT-based quantitative phosphoproteomes for drug-resistant cancer cells were downloaded from PRIDE (PXD007145 and PXD002735).
The reference proteome of human and mouse were downloaded from UniProt.
The reference genome of human and mouse were downloaded from Ensembl (release version 85 and 99, http://www.ensembl.org/).
The experimentally characterized human and mouse p-sites were taken from 9 public phosphorylation databases, including UniProt, SysPTM 2.0, PhosphoSitePlus, PhosphoPep 2.0, PHOSIDA, HPRD 9, Phospho.ELM 9.0, dbPTM 3.0 and dbPAF.
GO annotations were downloaded from the QuickGO (https://www.ebi.ac.uk/QuickGO/, on 21 October 2017).
KEGG annotations were downloaded from the ftp server of Kyoto Encyclopedia of Genes and Genomes (KEGG) (ftp://ftp.bioinformatics.jp/, released on 15 October 2017).
PK genes of human and mouse were taken from iEKPD 2.0 (http://iekpd.biocuckoo.org/).
TF-binding sites (TFBSs) of all available TFs were taken from 'Genome Tracks' of JASPAR database (http://expdata.cmmt.ubc.ca/JASPAR/downloads/UCSC_tracks/2018/hg38/).
known TF-target relations in H. sapiens were downloaded from the database of TRRUST (v2, http://www.grnpedia.org/trrust/).
The RNA-seq data has been deposited into NCBI Gene Expression Omnibus (GEO, https://www.ncbi.nlm.nih.gov/geo/) with the dataset identifier GSE169702. The mass spectrometry phosphoproteomic and proteomic data including the annotated mass spectra have been deposited into the integrated proteome resources (iProX, http://www.iprox.org/) with the dataset identifier PXD035829. All the other data supporting the findings of this study are available within the article and its supplementary information files. Source data are provided with this paper.

Field-specific reporting

Please select the one below that is the best fit for your research. If you are not sure, read the appropriate sections before making your selection.

☒ Life sciences ☐ Behavioural & social sciences ☐ Ecological, evolutionary & environmental sciences

For a reference copy of the document with all sections, see nature.com/documents/nr-reporting-summary-flat.pdf

Life sciences study design

All studies must disclose on these points even when the disclosure is negative.

Sample size	No sample-size calculation was performed. Sample sizes were chosen based on experience and published studies in the field (GUT, 2021, 70 (3):567-574; Protein Cell, 2020; 11(7):518–524; Nature Neuroscience, 2018: 21, 440-446; Hepatology, 2017; 66(6), 2002-2015)
Data exclusions	No data were excluded from analyses.
Replication	Each value reported the mean ± s.d. of at least three independent experimental replications, which are either independent experiment or individually treated cells.
Randomization	n/a. There are no experiment groups in this study for which samples needed to be allocated.
Blinding	The evaluations of the flow cytometry analyses were performed in a blinded manner. The groups were blinded for the investigators during analysis. No other experiments in this study required an analysis for which bias may alter the results.

Reporting for specific materials, systems and methods

We require information from authors about some types of materials, experimental systems and methods used in many studies. Here, indicate whether each material, system or method listed is relevant to your study. If you are not sure if a list item applies to your research, read the appropriate section before selecting a response.

Materials & experimental systems

n/a	Involved in the study
	☒ Antibodies
	☒ Eukaryotic cell lines
☒	Palaeontology and archaeology
	☒ Animals and other organisms
	☒ Human research participants
☒	Clinical data
☒	Dual use research of concern

Methods

n/a	Involved in the study
☒	ChIP-seq
	☒ Flow cytometry
☒	MRI-based neuroimaging

Antibodies

Antibodies used	Primary antibodies:<br>anti-GAPDH (Proteintech, 10494-1-AP)<br>anti-Pim-1 (D8D7Y) Rabbit mAb (Cell Signaling, 54523S)<br>anti-c-Myc antibody [Y69] (Abcam, ab32072)<br>anti-c-Myc (phospho S62) (Abcam,ab51156)<br>anti-4E-BP1 (53H11) (Cell signaling, 9644T)<br>anti-p-4E-BP1(T32/46) (Cell signaling, 2855T)<br>anti-E-cadherin (Invitrogen, 13-1900)<br>anti-Human Albumin cross adsorbed (Bethy,A80-229A)<br>anti-Albumin (GeneTex, GTX102419)<br>Human Serum Albumin APC-conjugated Antibody (R&D,IC1455A)<br>anti-rabit IgG, HRP-linked Antibody (Beyotime,A0208)<br>Cy3-conjugated AffiniPure Donkey Anti-Rabbit IgG (H+L) (min X Bov,Ck,Gt,GP,Sy Hms,Hrs,Hu,Ms,Rat,Shp Sr Prot) (Jackson,711-165-152)<br>Alexa Fluor™ 488 Donkey anti-Rat IgG (H+L) (Invitrogen, A-21208)<br>Alexa Fluor™ 568 Donkey anti-Rabbit IgG (H+L) (Invitrogen, A10042)
Validation	All primary antibodies are standard regents in biology study and obtained from commercial source. The quality control were performed by the relevant manufacturer as listed. The validation information for primary antibodies could be found: anti-GAPDH (Proteintech, https://www.ptglab.com/products/GAPDH-Antibody-10494-1-AP.htm); anti-PIM-1 (Cell Signaling, https://www.cellsignal.com/products/primary-antibodies/pim-1-d8d7y-rabbit-mab/54523); anti-c-Myc (Abcam, https://www.abcam.com/c-myc-antibody-y69-chip-grade-ab32072.html); anti-c-Myc (phospho S62) (Abcam, https://www.abcam.com/c-myc-phospho-s62-antibody-ab51156.html); anti-4E-BP1 (53H11) (Cell signaling, https://www.cellsignal.com/products/primary-antibodies/4e-bp1-53h11-rabbit-mab/9644); anti-p-4E-BP1 (T32/46) (Cell signaling, https://www.cellsignal.com/products/primary-antibodies/phospho-4e-bp1-thr37-46-236b4-rabbit-mab/2855); anti-E-cadherin (Invitrogen, https://www.thermofisher.cn/cn/zh/antibody/product/E-cadherin-Antibody-clone-ECCD-2-Monoclonal/13-1900); anti-Albumin (GeneTex, https://www.genetex.com/Product/Detail/Albumin-antibody/GTX102419); Human Serum Albumin APC-conjugated Antibody (R&D,https://www.rndsystems.com/cn/products/human-serum-albumin-apc-conjugated-antibody-188835_ic1455a)

Eukaryotic cell lines

Policy information about cell lines

Cell line source(s)	293FT (Invitrogen, R70007)<br>U-2 OS (American Type Culture Collection, HTB-96)<br>HDF (Fudan University)
Authentication	None of the cell lines were authenticated in this study.
Mycoplasma contamination	All cell lines were tested negative for mycoplasma contamination.
Commonly misidentified lines (See ICLAC register)	None

Animals and other organisms

Policy information about studies involving animals; ARRIVE guidelines recommended for reporting animal research

Laboratory animals	C57BL/6J, male, 8 weeks old
Wild animals	Not used.

Field-collected samples	Not used.
Ethics oversight	All animal experiment procedures were conducted in compliance with the approval of the Animal Ethics Committee at ShanghaiTech University (20200713002).

Note that full information on the approval of the study protocol must also be provided in the manuscript.

Human research participants

Policy information about studies involving human research participants

Population characteristics	Human adult fibroblasts were derived from skin biospies of two 35-year-old healthy male donors.
Recruitment	Donor recruitment and registration are organized by Eye & ENT Hospital, Fudan University. Only healthy individuals were recruited in this study.
Ethics oversight	The ethical committees of Eye & ENT Hospital, Fudan University approved all procedures of the collection of human skin biospies (2020035, 2021007-1). All participants have given informed consent.

Note that full information on the approval of the study protocol must also be provided in the manuscript.

Flow Cytometry

Plots

Confirm that:

- [x] The axis labels state the marker and fluorochrome used (e.g. CD4-FITC).
- [x] The axis scales are clearly visible. Include numbers along axes only for bottom left plot of group (a 'group' is an analysis of identical markers).
- [x] All plots are contour plots with outliers or pseudocolor plots.
- [x] A numerical value for number of cells or percentage (with statistics) is provided.

Methodology

Sample preparation	For cell death analysis, cells were collected at day 5 after FHH or GFP transfection. Dead cells were detected by Annexin V-PE/7-AAD Apoptosis Detection Kit (Yeasen) according to the manufacturer's instructions.<br>For intracellular staining of ALBUMIN, 1×106 cells were harvested and fixed with 4% PFA for 30 min, and then permeabilized in with 0.25% Triton X-100 in 3% BSA for 10 min. The cells were then incubated with 3% BSA 1 hour at room temperature followed by incubating with primary antibody (anti-albumin, Bethyl) for 30 min in 3% BSA. Then, the cells were washed three times with cold PBS.<br>For EdU positive cells detection, Cells were incubated with 10 μM EdU culture medium (Reagent A,RIBOBIO, C10338-1, 1:5000 diluted in culture medium) for 24h at 37 degree. Then, the cells were washed with PBS two times (5 min/time) and fixed with 4% PFA for 30 min at room temperature, followed by incubation in 2 mg/ml glycine for 5 min on bleaching shaker. The cells were then washed 3 times in PBS (5 min/wash), and permeated cell membrane by 0.5%-Triton in PBS and incubate at room temperature for 10 min. Then, the cells were washed in PBS and incubated with 1× Apollo® dyeing reaction solution (Reagent B, C, D, E, RIBOBIO, C10338-1) for 30 minutes in the dark, room temperature, and bleaching shaker. After discarding the dyeing reaction solution, cells were incubated with 0.5%-Triton in PBS two times in dark (10 min/incubate). Then, the cells were incubated with 1× Hoechst 33342 (Reagent F, RIBOBIO, C10338-1, 1:100 diluted in distilled water) for 30 min in the dark, room temperature, and bleaching shaker. After incubation, the cells were then washed with PBS three times (5 min/time).
Instrument	BD LSRFortessa
Software	Data collected was analyzed by FlowJo
Cell population abundance	Abundance of interest cell populations was determined using negative control.
Gating strategy	Cell Debris was excluded by FSC-A/SSC-A. Doublets were excluded using FSC-A/ FSC-W gates. Remaining cells were analyzed using displayed markers. The gating strategy was shown in supplementary figure 8e.

- [x] Tick this box to confirm that a figure exemplifying the gating strategy is provided in the Supplementary Information.
